# Increased Risk of Drug-Induced Hyponatremia during High Temperatures

**DOI:** 10.3390/ijerph14070827

**Published:** 2017-07-22

**Authors:** Anna K. Jönsson, Henrik Lövborg, Wolfgang Lohr, Bertil Ekman, Joacim Rocklöv

**Affiliations:** 1 Department of Forensic Genetics and Forensic Toxicology, National Board of Forensic Medicine, Linköping 587 58, Sweden; 2 Department of Clinical Pharmacology, Department of Medical and Health Sciences, Linköping University, Linköping 581 85, Sweden; Henrik.Lovborg@regionostergotland.se; 3 Department of Public Health and Clinical Medicine, Epidemiology and Global Health, Umeå University, Umeå 901 85, Sweden; wolfgang.lohr@umu.se (W.L.); joacim.rocklov@umu.se (J.R.); 4 Department of Endocrinology, Department of Medical and Health Sciences, Linköping University, Linköping 581 85, Sweden; Bertil.Ekman@regionostergotland.se; 5 Institute of Public Health, Heidelberg University, Heidelberg 691 20, Germany

**Keywords:** average daily temperature, hyponatremia, adverse drug reaction

## Abstract

*Purpose*: To investigate the relationship between outdoor temperature in Sweden and the reporting of drug-induced hyponatremia to the Medical Products Agency (MPA). *Methods*: All individual adverse drug reactions (ADR) reported to MPA from 1 January 2010 to 31 October 2013 of suspected drug-induced hyponatremia and random controls were identified. Reports where the ADR had been assessed as having at least a possible relation to the suspected drug were included. Information on administered drugs, onset date, causality assessment, sodium levels, and the geographical origin of the reports was extracted. A case-crossover design was used to ascertain the association between heat exposure and drug-induced hyponatremia at the individual level, while linear regression was used to study its relationship to sodium concentration in blood. Temperature exposure data were obtained from the nearest observation station to the reported cases. *Results*: During the study period, 280 reports of hyponatremia were identified. More cases of drug-induced hyponatremia were reported in the warmer season, with a peak in June, while other ADRs showed an opposite annual pattern. The distributed lag non-linear model indicated an increasing odds ratio (OR) with increasing temperature in the warm season with a highest odds ratio, with delays of 1–5 days after heat exposure. A cumulative OR for a lag time of 1 to 3 days was estimated at 2.21 at an average daily temperature of 20 °C. The change in sodium per 1 °C increase in temperature was estimated to be −0.37 mmol/L (95% CI: −0.02, −0.72). *Conclusions*: Warm weather appears to increase the risk of drug-induced hyponatremia.

## 1. Introduction

Heat waves have been associated with increased mortality, especially in the elderly population [1,2]. During heat waves, an increased prevalence of hyponatremia in hospitalized patients has been observed [3,4] as well as increased mortality among patients with hyponatremia [5,6].

Hyponatremia is a known adverse drug reaction (ADR) to a number of medications, especially thiazide diuretics, antidepressants, and anticonvulsants [7,8]. The involvement of these drugs indicates that dehydration and sodium loss in combination with a syndrome of inappropriate antidiuretic hormone secretion (SIADH) represents the main pathophysiological causes for hyponatremia during unexpected warm periods Moreover, recommendations of excessive water intake during heat waves potentially create even more severe hyponatremia by dilution [9].

Whether drug-induced hyponatremia can explain excess mortality related to heat exposure at the population level is, however, not well studied. Compared to the general population, users of psychotropic drugs have been reported to have an increased risk of heat-related hospitalizations during a single heat wave, in a study performed in south western France (containing a temperate climate) [10]. Another study from China (containing a subtropical climate) could not find any seasonal variations in thiazide-induced hyponatremia disorders [11].

The aim of the present study was to investigate the relationship between outdoor temperature in Sweden (temperate climate) and reporting by healthcare professionals of drug-induced hyponatremia to the Swedish Medical Products Agency (MPA).

## 2. Method

Within the Pharmacovigilance system in Sweden, information on all ADRs reported to the MPA were stored in a national database; the Swedish Drug Information System (SWEDIS) until October 2013. Each ADR was classified with respect to its causality using the following World Health Organization (WHO) definition [12] Data stored in the database consisted of information about the patient, drugs used, suspected ADRs, outcome, causality assessment, origin of the report, and administrative data. It was also possible for the reporter to add information in a free text on e.g., laboratory findings. In SWEDIS, drugs could be listed as being suspected of having caused or contributed to the reaction, as interacting with another prescribed drug, or as concomitant medication not related to the ADR. Each ADR was classified using WHO definitions with respect to its causality [13].

All individual ADR reports from 1 January 2010 to 31 October 2013 where hyponatremia was suspected, were identified in SWEDIS. Only reports where the ADR was assessed as having a certain, probable or possible relation to the suspected drug, were included. Information on reported ADR (administered drugs, onset date, causality assessment and sodium levels) and the origin of the report (based on the address where the reporter worked) was extracted from the ADR reports. A total of 280 reports of ADRs, other than hyponatremia, were randomly selected in SWEDIS as controls during the study period.

Meteorological data was obtained from the open data repository at the Swedish Meteorological and Hydrological Institute (SMHI; http://opendata-catalog.smhi.se). The location of the case reports was linked to temperature exposure data at the closest nearby meteorological monitoring station. Average daily temperature readings were obtained for the onset date, as well as for the same day of Week one, and two weeks before and after that date. No temperature recording data was found to be missing for the days of the study.

### 2.1. Statistical Analysis

Data were formatted according to a time stratified case-crossover design using the same day of Week one, and two weeks before and after the case date as reference times. Due to the hypotheses relating to heat exposure, we made analyses looking at the whole year, but also excluded the winter period and focused our analysis on the period from May to September in analyses specifically researching heat-related morbidity. A conditional logistic regression model was fitted to compare the temperature exposure on the case days to the temperature exposure on the reference days. Initially delays between temperature exposure and events were investigated establishing temperature lag relationships to ADR events up to 10 days using the DLNM (Distributed Lag Non-Linear Models) package in the R statistical computing software [14]. The DLNM packages fits distributed non-linear lag models that are flexible and can easily describe complex relationships and latencies between exposure and event. Cubic B-splines were fitted using two degrees of freedom for temperature and lag dimensions respectively. Additional linear regression analysis assessed the association between the lowest sodium concentrations in blood in relationship to temperature. Delays between temperature exposure and physiological recordings were included according to the findings from the DLNM analysis. Information on the minimum sodium concentrations was missing for 36 cases.

### 2.2. Ethical Considerations

The project was approved at the regional ethical review board at Linköping University, Linköping, Sweden (Dnr: 2014/85-31).

## 3. Results

**Figure 1:**
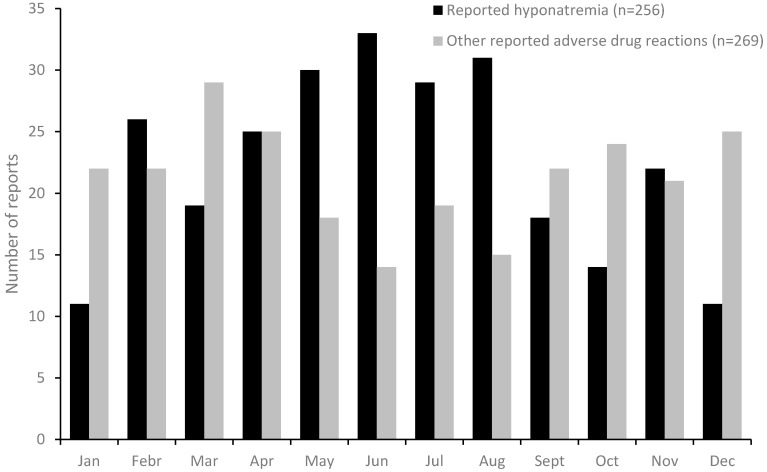
Annual seasonal pattern of reported hyponatremia and randomly collected control reports with other adverse drug reactions, in Sweden.

During the study period, 280 reports of hyponatremia was identified in SWEDIS. In these reports, and in the corresponding 280 randomly collected control reports with other ADRs, information on the month of onset was available in 256 and 269 cases respectively. Hyponatremia showed a clear seasonal pattern, with more reports in the warmer season, and a peak in June, while other ADR’s showed an opposite annual pattern (Figure 1).

**Figure 2:**
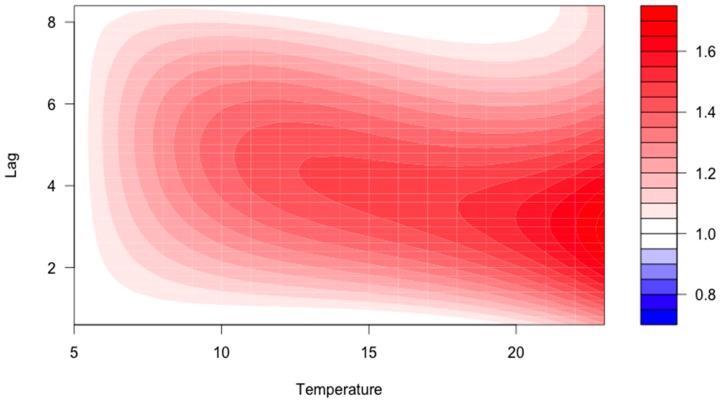
Contour graph describing the association between average daily temperature at different lag times and the corresponding odds ratio (OR; colored scale to the right) for drug-induced hyponatremia according to the blue to red color contrast.

Of the 280 included reports, 53 cases were excluded since geographical origin, onset of ADR, or weather data was missing. The remaining 125 reports with an ADR onset during summer (May–September) were linked to meteorological data. Among those, 95 were women (76%) and 30 were men (24%). The median age was 80 years (range 33–97 years). The case-crossover model suggested an increasing odds ratio (OR) with higher temperatures in the warm season with a latency of 1–5 days after the heat exposure (Figure 2). A cumulative OR (adding the independent associations) at a lag time of 1 to 3 days was 2.21 (non-significant) at an average daily temperature of 20 °C.

Supporting this finding, we found that when the daily average temperature had been increasing five days earlier; the sodium measurement in serum among subjects with ADR was statistically proportionally lower. The change in sodium per 1 °C increase in temperature was estimated to be −0.37 mmol/L (95% CI: −0.02, −0.72).

## 4. Discussion

We found increasing risks for reported drug-induced hyponatremia with higher temperatures during the warm season, and physiological evidence of reduced sodium concentrations with increasing temperature. The findings show empirical evidence of heat mechanisms of importance for preventing ill health during heat waves in the elderly. Prior studies have speculated potential mechanisms [2,10,15,16], but the lack of good registers of ADRs have limited the possibilities for empirical studies.

A previous study in the same region identified prior cardiovascular and mental disease as risk factors for mortality during heat exposure and heat waves [17]. The current study emphasized the importance of researching how drug use contributed to these deaths, in order to design successful public health interventions.

In a previous study [18], reporting of ADRs during two years of heatwaves (2003 and 2006) were compared with reporting during two reference years (2004 and 2005) in France. In that study, metabolic ADRs were less commonly reported during the years of heat waves compared to the reference period, otherwise the reported ADRs were similar between the periods. There was however, significantly more reports on ADRs associated with diuretics, serotonergic antidepressants, Angiotensin Converting Enzyme inhibitors (ACE inhibitors) and Proton Pump Inhibitors (PPIs) during heatwaves.

Human adaption to heat is dependent on the body’s ability to act as a cooling system where sweat production is the primary physiological way to lose heat [2]. Hyponatremia may be ascribed to either water retention or loss of effective solute (sodium plus potassium) in excess of water. Hyponatremia is mostly related to an excess of antidiuretic hormone (ADH) [19]. Hyponatremia has been reported during heavy and prolonged endurance exercise such as marathons, which is a result of excessive sweating, combined with a large intake of mainly hypotonic drinks [20]. A warm climate in summer may cause a similar situation, which is compensated for in most healthy individuals. Drugs may affect the sodium hemostasis and water hemostasis directly (e.g., diuretics) but also ADH secretion centrally (e.g., psychotropics) [7], thereby increasing the risk of hyponatremia during warm weather, as observed in this study. A potential contributing factor to the relationships found in this study may be exacerbated recommendations of water intake (but not salt) during heat exposure, which may negatively affect people who use drugs, leading to hyponatremia, and which can also explain the latency in the relationship [9].

### Strengths and Limitations

The main strength of this study was the population-based design; all cases reported to the MPA in Sweden were included in the study. For cases with geographical origins, we had a complete set of meteorological data regarding temperature. A strength of the study was also the self-controlled matched design which reducing confounding by traditional confounding factors at an individual level.

The main limitation was that the study was based on spontaneously reported ADRs where underreporting is a well-known problem [21]. Hence, not all diagnosed hyponatremias were included, but only those that were reported by healthcare staff. Furthermore, the lack of date of onset of hyponatremia or geographical location of the report was missing in some cases, limiting the number of cases available to analyze the relation between hyponatremia and heat exposure.

Based on the information reported, it was not possible to distinguish other factors that could have contributed to the hyponatremia. To minimize this problem, we included only the reports that were assessed as at least possibly related to the suspected drug intake. The underlying disease and other factors could be have been the cause of, or a contributing factor to the symptoms. We could not review possible differences between groups of drugs in relation to heat and drug-induced hyponatremia, due to the low number of reports.

In this study, it was not possible to discriminate between the heat and drug exposures as a cause of the hyponatremia. However, the increasing levels of reports with higher temperatures and the attribution of hyponatremia to medication use suggested an interaction between the two factors. Based on the nature of the data source, only drug-treated patients with hyponatremia were included.

Despite our study population being small, and larger studies being necessary to confirm our findings, the results shown are to be taken seriously, and the effects of hyponatremia can likely be avoided by modifying medication use and behavior during heat exposure. The results further suggest that even in temperate climates, with relatively moderate high temperature, ambient heat exposure can interact with drugs and cause hyponatremia.

## 5. Conclusions

Warm weather increases the risk of drug-induced hyponatremia. Increased awareness of risk drugs and adjustment of medication use by patients with a high risk of drug-induced hyponatremia could potentially prevent this condition.
